# Whole genome sequencing of HER2-positive metastatic extramammary Paget’s disease: a case report

**DOI:** 10.1186/s13023-024-03169-y

**Published:** 2024-06-03

**Authors:** Boon Yee Lim, Zexi Guo, Jing Quan Lim, Tun Kiat Ko, Elizabeth Chun Yong Lee, Bavani Kannan, Jing Yi Lee, Abner Herbert Lim, Zhimei Li, Cedric Chuan-Young Ng, Inny Busmanis, Jason Yongsheng Chan

**Affiliations:** 1 Cancer Discovery Hub, National Cancer Centre Singapore, Singapore, Singapore; 2 Division of Cellular and Molecular Research, National Cancer Centre Singapore, Singapore, Singapore; 3 Duke-NUS Medical School, Singapore, Singapore; 4 Department of Anatomical Pathology, Singapore General Hospital, Singapore, Singapore; 5 Divison of Medical Oncology, National Cancer Centre Singapore, Singapore, Singapore

**Keywords:** Precision oncology, Targeted therapy, Next generation sequencing, ERBB2, Trastuzumab

## Abstract

**Background:**

Extramammary Paget’s disease (EMPD) is a rare cancer that occurs within the epithelium of the skin, arising predominantly in areas with high apocrine gland concentration such as the vulva, scrotum, penis and perianal regions. Here, we aim to integrate clinicopathological data with genomic analysis of aggressive, rapidly-progressing *de novo* metastatic EMPD responding to HER2-directed treatment in combination with other agents, to attain a more comprehensive understanding of the disease landscape.

**Methods:**

Immunohistochemical staining on the scrotal wall tumor and bone marrow metastasis demonstrated HER2 overexpression. Whole genome sequencing of the tumor and matched blood was performed.

**Results:**

Notable copy number gains (log_2_FC > 0.9) on chromosomes 7 and 8 were detected (*n* = 81), with 92.6% of these unique genes specifically located on chromosome 8. Prominent cancer-associated genes include *ZNF703*, *HOOK3*, *DDHD2*, *LSM1*, *NSD3*, *ADAM9*, *BRF2*, *KAT6A* and *FGFR1.* Interestingly, *ERBB2* gene did not exhibit high copy number gain (log_2_FC = 0.4) although 90% of tumor cells stained HER2-positive. Enrichment in pathways associated with transforming growth factor-beta (TGFβ) (FDR = 0.0376, Enrichment Ratio = 8.12) and fibroblast growth factor receptor (FGFR1) signaling (FDR = 0.0082, Enrichment Ratio = 2.3) was detected. Amplicon structure analysis revealed that this was a simple-linear amplification event.

**Conclusion:**

Whole genome sequencing revealed the underlying copy number variation landscape in HER2-positive metastatic EMPD. The presence of alternative signalling pathways and genetic variants suggests potential interactions with HER2 signalling, which possibly contributed to the HER2 overexpression and observed response to HER2-directed therapy combined with other agents in a comprehensive treatment regimen.

**Supplementary Information:**

The online version contains supplementary material available at 10.1186/s13023-024-03169-y.

## Background

Extramammary Paget’s disease (EMPD) is a rare adenocarcinoma typically presenting as an intraepithelial carcinoma [1]. Areas with high apocrine gland concentration such as the vulva, scrotum, penis, perineum, perianal region and axillae are predominantly affected [1]. Although there is favourable prognosis for the early disease stage, in certain cases, it can advance to invasive EMPD. Invasive EMPD is characterized by infiltration into deeper tissues and is associated to nodal and distant metastasis [1]. EMPD usually occurs in elderly individuals aged from 60 to 80 years, with an annual incidence ranging from 0.1 to 2.4 new cases per million people [1]. Only about 20% of patients with EMPD present with distant metastatic disease [1]. As a result, there is little guidance on treatment strategies, resulting in dismal outcomes [2].

Managing EMPD is complex due to the lack of standardized treatment protocols [3]. Surgical intervention, such as wide local excision or radical vulvectomy, is common for localized disease, yet recurrence rates range from 20 to 70% [3]. Invasive cases may require inguinofemoral lymphadenectomy or sentinel lymph node biopsy, while non-surgical options like radiation therapy (RT) are considered for unresectable, recurrent, or metastatic disease [3]. Despite these treatment modalities, improving patient outcomes through personalized targeted therapy continues to pose a challenge.

EMPD share similar histological and clinical features as Mammary Paget’s Disease (MPD) [4]. MPD is a skin condition marked by eczematous lesions usually presenting on the nipple or areolar regions and is frequently linked to underlying in situ or invasive breast cancer [4]. Human epidermal growth factor receptor 2 (HER2) is a transmembrane receptor tyrosine kinase encoded by the *ERBB2* gene. Within the epidermal growth factor receptor family, HER2 is widely recognized for its oncogenic role in a subset of invasive breast cancers [5]. In MPD, *ERBB2* amplification and consequent HER2 overexpression has been demonstrated in up to 90% of the cases [6, 7]. In EMPD, however, the frequency of HER2 overexpression has been generally inconsistent, with small-scale studies reporting lower frequencies of HER2 overexpression ranging from 15 to 65% [7–10], while *ERBB2* gene amplification ranged from 13 to 43% [8, 11–13]. Despite the anecdotal occurrences of metastatic EMPD cases, many studies have posited a correlation between HER2 overexpression and the invasive nature of EMPD [7, 9, 12, 13]. Recent advancements made towards developing HER2-targeted therapies have greatly expanded the treatment options of HER2-expressing breast cancer [11, 13]. Such HER2-directed approaches have also been described in a few EMPD cases [2, 14–16]. However, the treatment efficacy has yet to be ascertained given differing patient responses within the limited number of cases studied. Furthermore, none of the studies have described the clinical observations in the context of the underlying tumor genomic landscape.

Herein we present a case report of a patient with *de novo* metastatic EMPD who responded rapidly to HER2-directed therapy combined with other agents in a comprehensive treatment regimen. Whole genome sequencing (WGS) revealed a distinctive copy number landscape with potential clinical implications.

## Materials and methods

### Patient data and biospecimen collection

All clinical information was retrieved from electronic medical records. Verification of demographic data including sex, age and ethnicity of the affected patient was corroborated by National Registry Identification Card. All immunohistochemistry was performed at the pathology services laboratory of the Singapore General Hospital and histological parameters were reviewed by an expert dermatopathologist (B.I.).

### Whole genome sequencing (WGS) and variant calling

DNA isolated from snap-frozen tumor tissue and matched whole blood was selected for whole-genome sequencing. Whole-genome sequencing was performed on the Illumina NovaSeq 6000 platform as paired-end 150 bp reads, using DNA inserts averaging 350 bp (NovogeneAIT Genomics). The raw sequencing data obtained from the WGS was subjected to a series of data pre-processing steps using the nextflow sarek workflow [17]. This workflow incorporates various tools and pipelines for quality control, alignment, and variant calling. The following steps were performed: Sequencing quality control (FastQC) [18]; Map Reads to Reference GRCh38 (GATK) (BWA mem) [19]; Mark Duplicates (GATK MarkDuplicates) [20]; Base (Quality Score) [21]; Recalibration (GATK BaseRecalibrator, GATK ApplyBQSR) [22]; Preprocessing quality control (samtools stats) [23]; Preprocessing quality control (mosdepth) [24]; Overall pipeline run summaries (MultiQC) [25]; variant calling (Mutect2) [26]; variant annotation (VEP) [27]. After annotating the variants, a rigorous selection process was applied to retain solely non-synonymous variants with deleterious effects, resulting in a refined subset of 43 variants.

### Mutational signatures

The mSigAct tool [28] was employed to investigate the presence and activity of mutational signatures within our WGS data. mSigAct employs a conservative maximum likelihood approach to determine the presence of specific mutational signatures within a spectrum. Additionally, it identifies the minimum subset of signatures necessary to reconstruct the observed spectrum. The sparse assign signatures functionality favors using the fewest signatures possible. It also enables maximum a posteriori estimation of signature activity by considering the proportion of tumors with a particular signature in a given type and the likelihood of a specific signature combination generating the observed spectrum.

### Copy number variations (CNV) analysis

Whole-genome sequencing data for the matched tumor-normal pairs was analyzed to detect copy number variations (CNVs) using CNVkit [29]. Based on the assumption that the normal sample has a diploid genome with no major copy number alterations, CNVkit compares the read counts in the cancer sample with those in the normal sample. The baseline for copy number calling on the tumor cohort was applied using the mean value of overall coverage. Copy counts were computed based on the depth of coverage in each location after GC bias and regional bias were corrected. The threshold of a notable CNV gain was set as log_2_FC > 0.9; likewise, a notable CNV loss was reported if log_2_FC < -0.9. Only genes that met the threshold of CNV gain and loss were considered for subsequent pathway enrichment analysis. The WEB-based GEne SeT AnaLysis Toolkit (Web Gestalt) tool was used for Over Representation Analysis (ORA) [30]. Enriched gene sets with a false discovery rate (FDR) of < 0.05 were considered significant. Pathway Enrichment via Functional Annotation Clustering was conducted with the Database for Annotation, Visualization, and Integrated Discovery (DAVID) [31]. Annotation clusters with Benjamini values < 0.05 were considered significant.

The median VAF was inferred from the SNV analysis. To obtain the purity-normalized absolute copy number, the sample was assumed to be copy-neutral (ploidy = 2) and a cutoff log2(copy_ratio) of 0.9 was applied to identify regions of significant amplification. AmpliconArchitect (AA) [32] was used to detect and characterize the amplicon structures. AA used an absolute copy number cutoff of 5 and a minimum span of 100 kilobase pairs (kbp) for the detection of any circular DNA structures that can carry amplified genetic material. This criterion was used to identify the seeding intervals and potentially seek out amplicon structures. The annotations provided by AA revealed the amplified genes associated with cancer within the identified amplicon. To further analyze the structure of amplicon, AA_classifier was used. Through the analysis, it was determined that the identified amplicon lacked cyclic edges, ruling out the possibility of extrachromosomal DNA (ecDNA) or associations to a break-fusion-bridge event and concluding that a simple-linear amplification event is observed in the sample.

TSGene, an evidence-based online repository dedicated to tumor suppressor genes (TSGs) [33], catalogs data pertaining to 1217 human TSGs (including 1018 genes and 199 long non-coding RNAs). Venn analysis was conducted on genes that met the specified threshold for CNV loss (log_2_FC < -0.9), revealing the subset of genes that shared overlap with known TSGs.

## Results

### Clinical case presentation

**Fig. 1:**
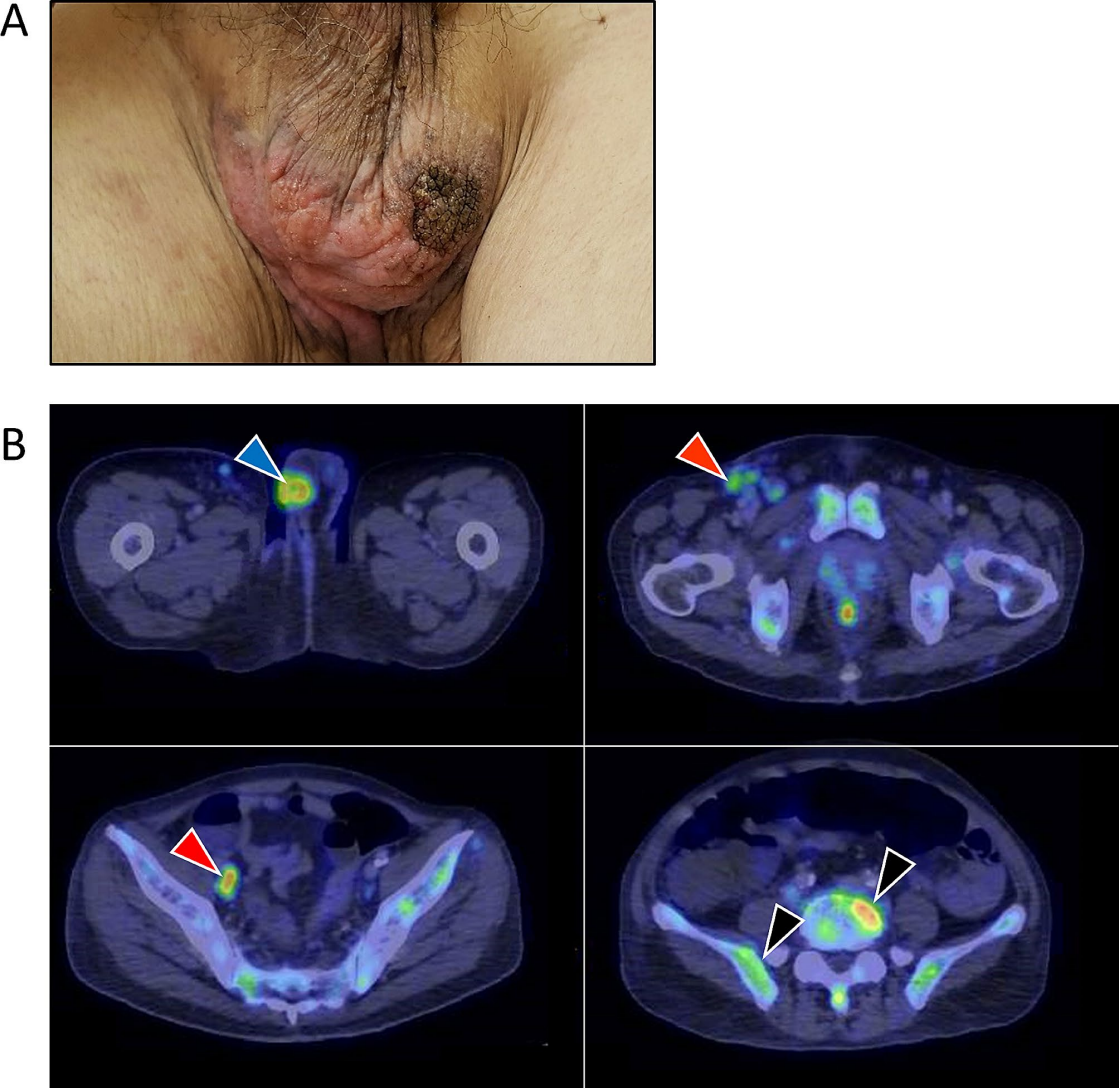
Clinical and diagnostic features of the patient with metastatic EMPD. **(A)** Erythematous hyperkeratotic rash with central ulceration over the scrotum. **(B)** PET/CT imaging. Blue arrow indicates an FDG-avid scrotal wall mass measuring 2.8 cm x 2.5 cm (SUVmax 6.9). Red arrows indicate right iliac and inguinal lymph nodes involvement (SUVmax 6.7). Black arrows indicate extensive FDG-avid mixed lytic-sclerotic bony lesions involving the ribs (not shown), spine and pelvis (SUVmax 9.3)

**Fig. 2:**
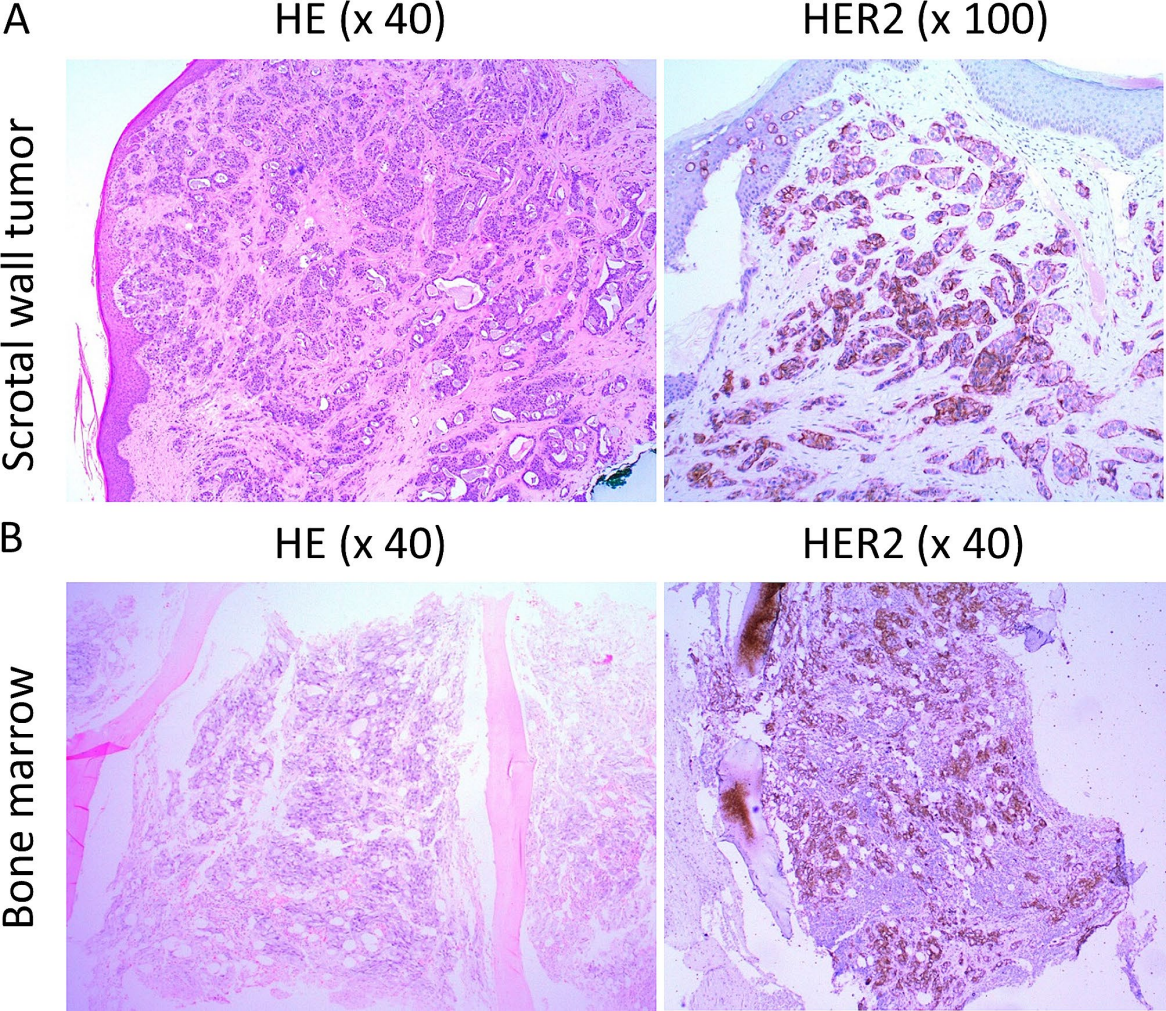
Immunohistochemical (IHC) staining of the scrotal wall tumor and bone marrow presenting HER2 positivity. **(A)** Representative image of HER2-positive scrotal wall tumor with strong membranous staining on 90% of tumor cells at 100X magnification. **(B)** Representative image of HER2-positive bone marrow showing HER2 staining on approximately 40% of cells, indicating a case of metastatic EMPD

The patient is a 68-year-old man of Chinese ethnicity, who initially presented with progressive low back pain associated with unintended weight loss of 5 kg over two months. Physical examination revealed an erythematous hyperkeratotic rash with central ulceration over the scrotum (Fig. 1A). He also had a fever measuring 38.6℃. Fluorodeoxyglucose-Positron-Emission Tomography/Computed Tomography (FDG-PET/CT) imaging showed an FDG-avid scrotal wall mass measuring 2.8 cm x 2.5 cm (Maximum Standardized Uptake Value, SUVmax 6.9), extensive FDG-avid mixed lytic-sclerotic bony lesions involving the spine and ribs (SUVmax 9.3), as well as the right iliac and inguinal lymph nodes (SUVmax 6.7) (Fig. 1B). Histologic examination of the scrotal skin showed thickened epidermis replaced by atypical epithelioid cells with enlarged hyperchromatic nuclei and abundant clear to eosinophilic or vacuolated cytoplasm, as well as regions of invasive carcinoma. Bone marrow biopsy showed moderate haematopoietic effacement. Primary tumor cells were diffusely positive for CK7, CK20, Cam5.2 and negative for SOX10, TTF1, PSA, and CDX2. Metastatic tumor cells exhibited a similar staining pattern. Additional immunostaining on the metastatic tumor was positive for GATA3, androgen receptor and estrogen receptor, but negative for progesterone receptor. HER2 was positive in over 90% of the tumors cells with at least 40% showing 3 + intensity (Fig. 2). Given the known association of Paget’s disease with the genitourinary and gastrointestinal tracts [34], oesophagogastroduodenoscopy and colonoscopy, as well as urine cytology were performed, all of which gave negative results for malignancy.

**Fig. 3:**
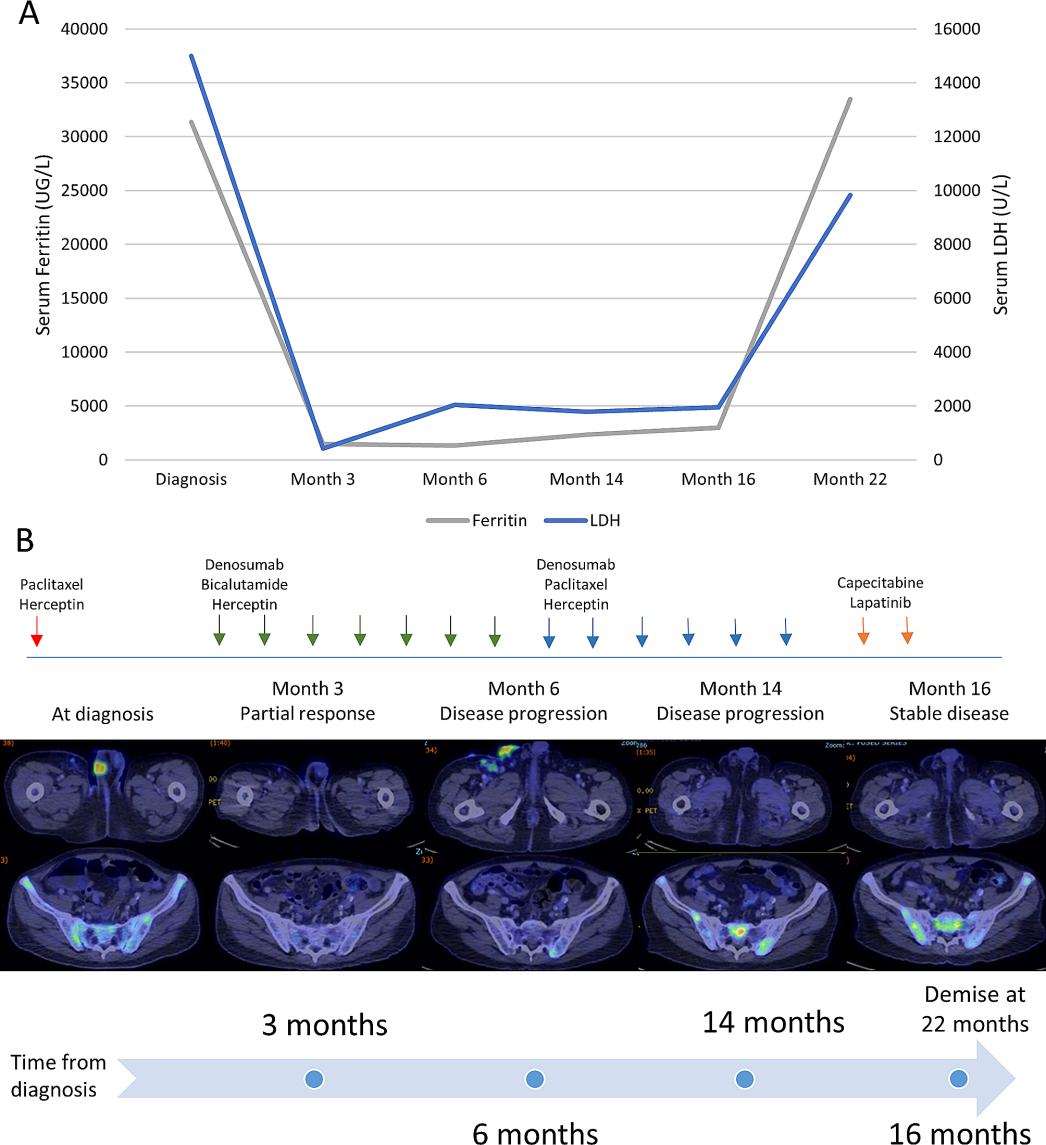
Overall timeline of patient’s disease history. **(A)** Serial monitoring of serum ferritin and LDH levels in the patient upon diagnosis and at months 3, 6, 14, 16 and 22, presenting changes in a potential serum biomarker levels over time. **(B)** Chronological representation of the patient’s PET/CT imaging results and drug therapy administered accordingly upon diagnosis and at months 3, 6, 14, and 16. Patient met with eventual demise at month 22 following disseminated metastases

Over the course of investigation, the patient developed an emergent syndrome indicative of rapid disease progression. Serial blood work over 14 days revealed worsening microangiopathic haemolytic anaemia (MAHA) with rapidly falling haemoglobin levels (nadir, 5.2 g/dL), platelet counts (nadir, 33 × 10^9^/L), raised serum lactate dehydrogenase (LDH) levels (from 1,908 to > 15,000 U/L), serum hyperferritinaemia (from 5,300 to 31,354 µg/L), decreased serum haptoglobin levels (< 0.10 g/L) and negative Direct Coomb’s test. The patient subsequently responded promptly to a combination of intravenous paclitaxel (80 mg/m^2^) and intravenous trastuzumab (8 mg/kg). Subcutaneous denosumab (120 mg) was administered on days 1, 8 and 15. Further doses of paclitaxel on days 8 and 15 were omitted due to the development of pneumonia requiring antibiotic therapy. By the time he was due for cycle 2 of treatment, ferritin (3,465 µg/L), LDH (983 U/L), haemoglobin (8.8 g/dL) and platelet counts (318 × 10^9^/L) had improved significantly. The patient was continued on HER2-directed therapy along with an anti-androgen (oral bicalutamide 150 mg daily, subcutaneous trastuzumab 600 mg and denosumab 120 mg once every 4 weeks) for another 7 cycles until disease progression (partial response at best). Subsequently, bicalutamide was replaced by paclitaxel (175 mg/m^2^, given once every 4 weeks) for a total of 6 cycles until disease progression, achieving partial response (PR) as best response. Thereafter, he was treated with capecitabine (1,000 mg twice daily for 2 weeks) and lapatinib (1,250 mg daily) given every 3-weeks for nine cycles (stable disease as best response), followed by a single dose of TDM-1 (no response). Then, the patient’s condition worsened with rapid elevation of ferritin and LDH levels, leading to his eventual demise about 22 months after diagnosis. The treatment strategy and serial PET/CT images are shown in Fig. 3.

### Copy number variants (CNV) analysis

**Fig. 4:**
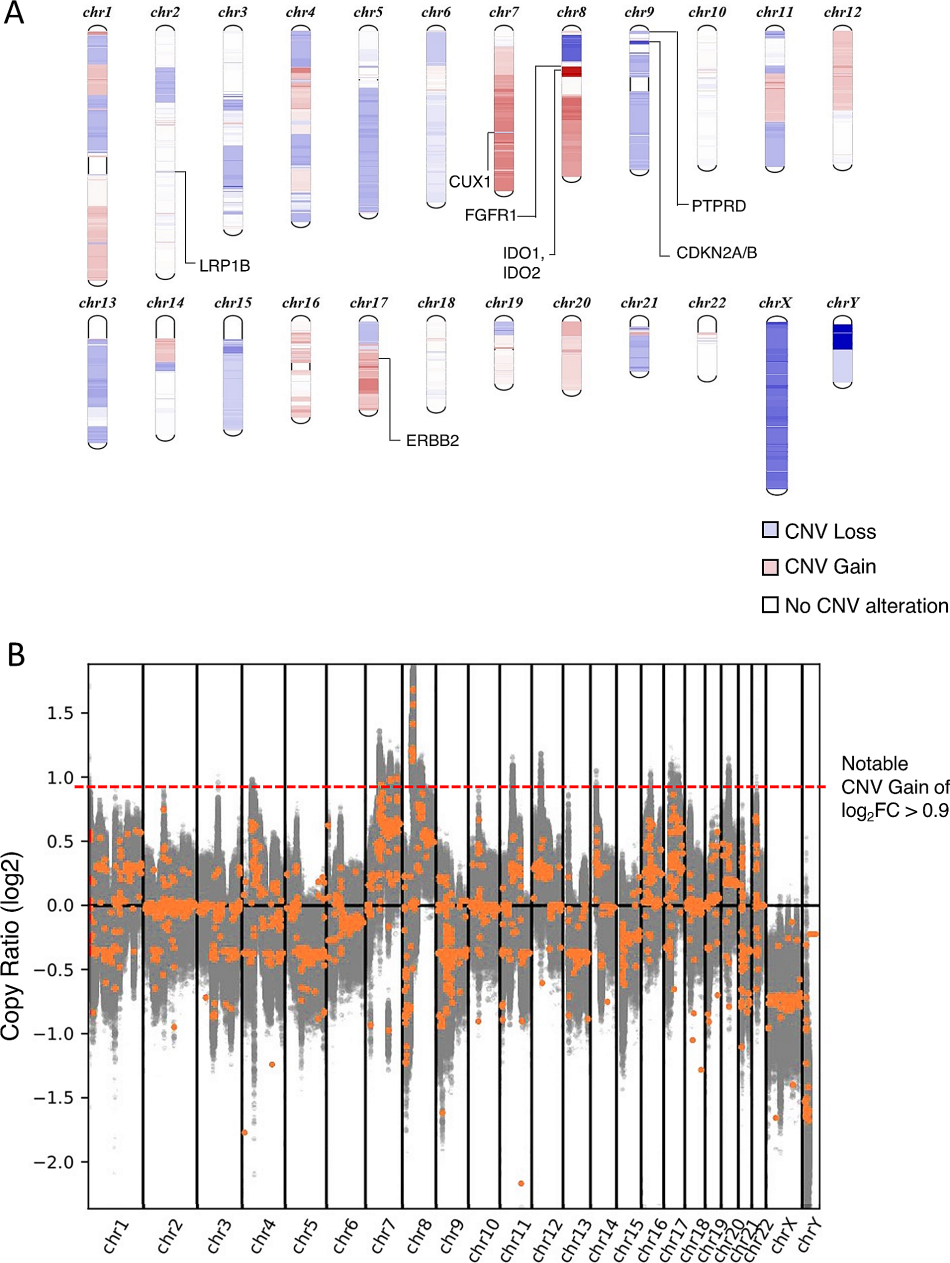
WGS was performed on the EMPD tumor and copy number alterations were analysed by CNVkit. **(A)** Schematic diagram of chromosomal regions exhibiting copy number variation (CNV) in the patient’s genome. Gene loci with copy number losses (Log2FC < 1) are denoted by blue bands located on the chromosome while those with copy number gains (Log2FC > 1) are denoted by red bands. Darker hues of blue indicate the presence of multiple gene loci with copy number losses within that chromosomal region; similarly, darker hues of red indicate the presence of multiple gene loci with copy number gains. **(B)** Copy numbers of the variants were presented using segmented log2 ratios (y-axis) on the respective chromosomal locations (x-axis). Gray spots indicate each variant and variants with indicative genes denote the orange spots. The orange spots above the dotted red line denote genes exhibiting notable CNV gains (log_2_FC value greater than 0.9), present on chromosome 7 and 8

**Fig. 5:**
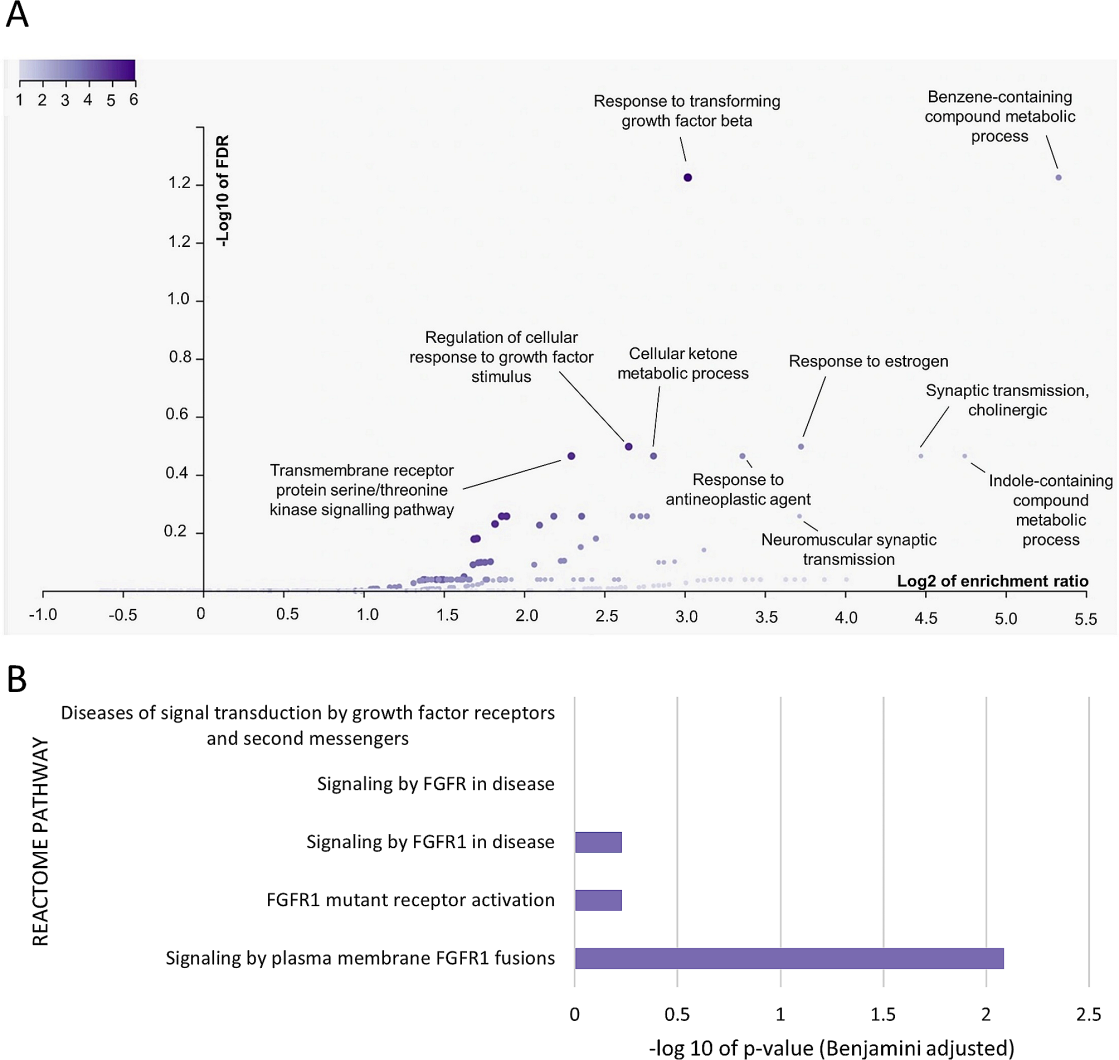
Functional enrichment analysis of genes in chromosome 8 with notable copy number gain. **(A)** Gene Ontology Over Representation Analysis (WebGestalt) of genes on chromosome 8 with log_2_FC > 0.9 reported an enrichment in the Response to TGFβ Pathway (GO:0071559, FDR = 0.0376, Enrichment Ratio = 8.12; genes involved: *ADAM9*, *FNTA*, *HTRA4*, *SFRP1*, *STAR*, *ZNF703*) and the Benzene-containing Compound Metabolic process (GO:0042537, FDR = 0.0376, Enrichment Ratio = 40.2; genes involved: *IDO1*, *IDO2*, *STAR*) **(B)** DAVID functional annotation clustering analysis identified a FGFR1 fusion pathway (REACTOME Pathway ID: R-HSA-8,853,336, FDR = 0.0082, Enrichment Ratio = 2.3; genes involved: *BAG4*, *ERLIN2*, *FGFR1*).

The WGS data revealed several chromosomal regions exhibiting copy number gains and losses in comparison to the reference normal sample. Among these regions, 81 unique genes on genomic loci chromosomes 7 and 8 demonstrated notable copy number gains (log_2_FC > 0.9), of which 92.6% of them (75 genes) are located on chromosome 8 (Fig. 4). Web-based databases were used to functionally annotate the 75 genes on chromosome 8 that harbored copy number gains (Table 1). This gene list showed an enrichment for the Response to transforming growth factor-beta (TGFβ) Pathway (GO:0071559, FDR = 0.0376, Enrichment Ratio = 8.12). Genes involved in the TGFβ pathway included *ADAM9*, *FNTA*, *HTRA4*, *SFRP1*, *STAR* and *ZNF703*. Additionally, these genes were enriched in a FGFR1 fusion signaling pathway (REACTOME Pathway ID: R-HSA-8,853,336, FDR = 0.0082, Enrichment Ratio = 2.3). The genes identified in this pathway were *BAG4*, *ERLIN2* and *FGFR1* (Fig. 5). Other significant pathways that were enriched include the Benzene-containing Compound Metabolic process (GO:0042537, FDR = 0.0376, Enrichment Ratio = 40.2). The genes involved are *IDO1*, *IDO2* and *STAR* (Fig. 5). *ERBB2* gene on chromosome 17 presented a copy number log_2_FC value of 0.4 despite strong HER2 protein overexpression observed from immunohistochemistry.

**Table 1:** List of genes (*n* = 81) with copy number change log2 FC ratio > 0.9. 75 out of the 81(92.6%) genes are located on Chromosome 8. Genes are ranked in descending order according to the log2 ratios

Chromosome	Gene Symbol	log_2_ FC
chr8	*SLC20A2*, *SMIM19*, *CHRNB3*, *CHRNA6*	1.67994
chr8	*THAP1*, *RNF170*, *MIR4469*, *HOOK3*, *FNTA*, *POMK*, *HGSNAT*, *POTEA*	1.56512
chr8	*IDO1*, *IDO2*, *TCIM*, *SIRLNT*, *ZMAT4*	1.22256
chr8	*SFRP1*, *MIR548AO*, *SNORD65B*, *GOLGA7*, *GINS4*, *LOC102723729*, *GPAT4*, *NKX6-3*, *ANK1*, *MIR486-1*, *MIR486-2*, *KAT6A*, *LOC105379393*, AP3M2, *PLAT*, *LOC101929897*, *IKBKB*, *POLB*, *DKK4*, *VDAC3*	1.21592
chr8	*UNC5D*, *KCNU1*, *LINC01605*, *ZNF703*, *LOC101929622*, *LOC102723701*, *ERLIN2*, *LOC728024*,*PLPBP*, *ADGRA2*, *BRF2*, *RAB11FIP1*, *GOT1L1*, *ADRB3*, *EIF4EBP1*, *ASH2L*, *STAR*, *LSM1*, *BAG4*, *DDHD2*, *PLPP5*, *NSD3*, *LETM2*, *FGFR1*, *C8orf86*, *RNF5P1*, *TACC1*, *PLEKHA2*, *HTRA4*, *TM2D2*, *ADAM9*, *SNORD38D*, *ADAM32*, *ADAM5*	1.20635
chr8	*ADAM3A*, *LOC100130964*, *ADAM18*, *ADAM2*	1.1306
chr7	*TCAF1*	0.99161
chr7	*DOCK4*, *ZNF277*	0.98376
chr7	*IMMP2L*	0.97464
chr7	*CT66*, *AUTS2*	0.94216

A tumor purity of about 44% was estimated using the median Variant Allele Frequency (VAF) from SNV analysis, resulting in a purity-normalized absolute copy number of 5.937. Among five seeding intervals identified on chromosomes 7 and 8 using the AmpliconArchitect (AA) tool, only one amplicon showed gene amplification, including *ZNF703, HOOK3, DDHD2, LSM1, WHSC1L1, ADAM9, BRF2, MYST3, and FGFR1*. These genes were also detected in the CNV analysis. The lack of cyclic edges in this amplicon suggests that the amplification event is a simple-linear one.

Genes with notable copy number loss (log_2_FC < -0.9) were primarily located on the X and Y sex chromosomes, as well as chromosomes 7, 8 and 9. Among these genes, *CDKN2A*, *CDKN2B*, *CUX1*, *LRP1B* and *PTPRD* were retrieved as tumor suppressors from the TSGene database.

### Somatic variants and mutational signatures

**Fig. 6:**
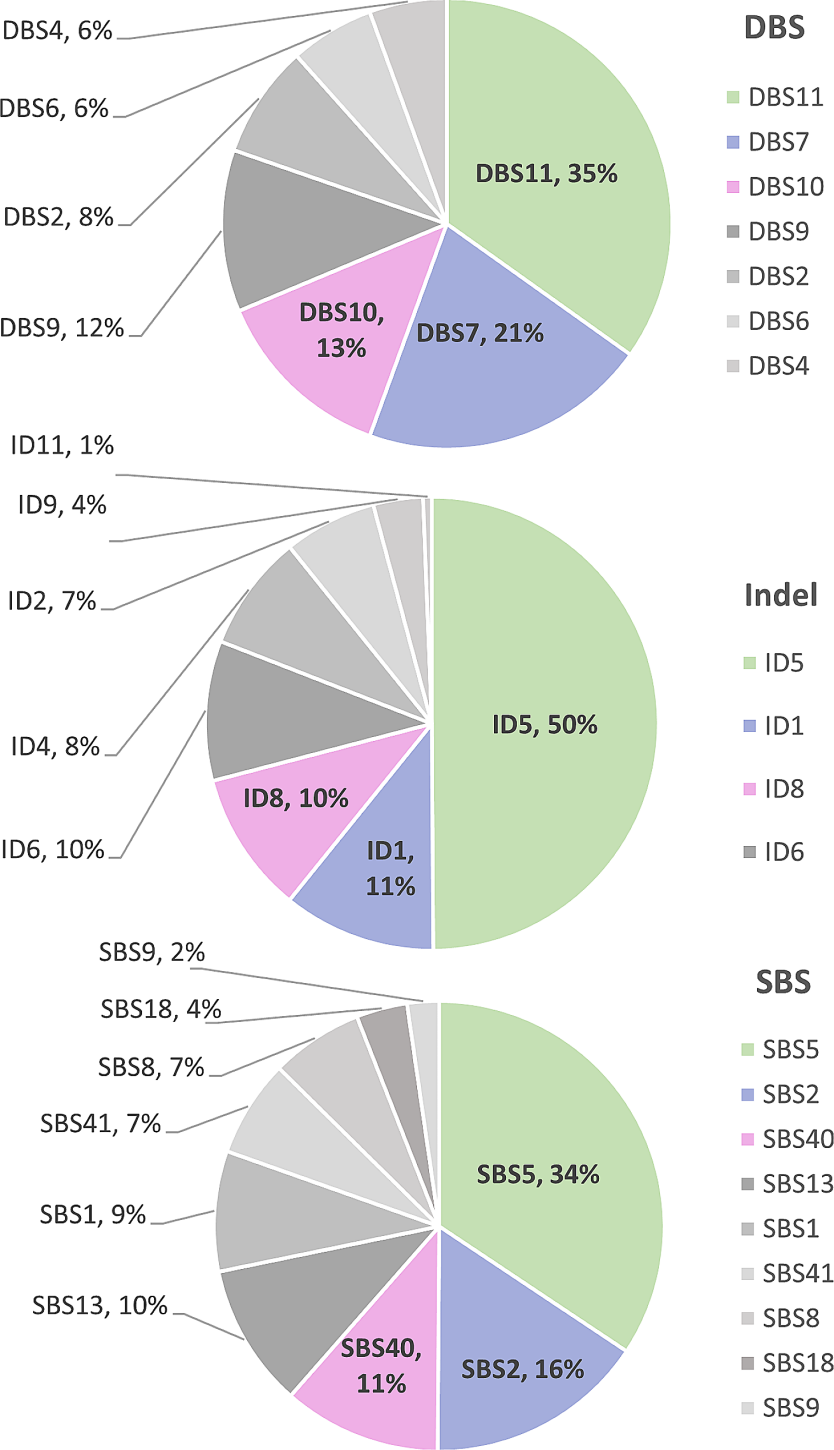
COSMIC mutational signatures identified in the case of EMPD. Proportion of SBS (Single Base Substitutions), ID (Insertion-Deletions) and DBS (Double Base Substitutions) mutational signatures from the WGS data of the EMPD tumor, as determined by mSigAct.

Variant calling analysis identified 43 genes harbouring non-synonymous variants with potentially deleterious effects (Table 2), including *PRAMEF2* and *PTPRD*. From WGS data, COSMIC mutational signatures were extracted, with 49.90%, 10.91%, and 10.13% of the data resembling COSMIC signatures ID5, ID1, and ID8, respectively. Additionally, 34.25%, 15.76% and 11.36% resembled signature SBS5, SBS2, and SBS40 respectively. For DBS signatures, 34.85%, 20.71% and 13.13% resembled DBS11, DBS7, and DBS10 respectively (Fig. 6). These signatures primarily relate to aging (ID5, ID1, ID8, SBS5, SBS40) and defective DNA repair (DBS7, DBS10), with SBS2 and DBS11 linked to APOBEC. SBS2’s co-occurrence with SBS13 [35] aligns with our findings; 10% of the data resembled SBS13.

**Table 2:** Summary of Single Nucleotide Variants (SNV) detected via Variant Calling analysis. Through an extensive selection process with the help of various variant filtering tools, a curated set of 43 genes harbouring non-synonymous mutations with deleterious effects was identified

Gene Symbol	Amino Acid Change	Consequence	Chromosome Number	SIFT Score	SIFT Impact	PolyPhen Score	PolyPhen Impact
*FSIP2*	p.Ile5606Met	Missense	2	0	Deleterious	0.117	Benign
*RLF*	p.Leu551His	Missense	1	0	Deleterious	0.266	Benign
*PTPRD*	p.Glu1510Lys	Missense	9	0	Deleterious	0.344	Benign
*OR4D10*	p.Met118Val	Missense	11	0	Deleterious	0.399	Benign
*PTPN21*	p.Pro447Ser	Missense	14	0	Deleterious	0.437	Benign
*MAPT*	p.Ser757Leu	Missense	17	0	Deleterious	0.532	Likely Damaging
*TBX18*	p.Ser550Phe	Missense	6	0	Deleterious	0.648	Likely Damaging
*MOCOS*	p.Ser362Tyr	Missense	18	0	Deleterious	0.696	Likely Damaging
*PLEKHM3*	p.Trp536Cys	Missense	2	0	Deleterious	0.831	Likely Damaging
*SFI1*	p.Leu929Phe	Missense	22	0	Deleterious	0.856	Likely Damaging
*PCDH18*	p.Glu740Val	Missense	4	0	Deleterious	0.915	Likely Damaging
*FBLN2*	p.Arg842Cys	Missense	3	0	Deleterious	0.927	Likely Damaging
*C2orf69*	p.Leu191Ser	Missense	2	0	Deleterious	0.935	Likely Damaging
*CEP162*	p.Lys1113Thr	Missense	6	0	Deleterious	0.935	Likely Damaging
*PLEKHA5*	p.Ser477Thr	Missense	12	0	Deleterious	0.977	Likely Damaging
*USH2A*	p.Tyr2960His	Missense	1	0	Deleterious	0.982	Likely Damaging
*INPP5F*	p.Lys168Asn	Missense	10	0	Deleterious	0.989	Likely Damaging
*PLEKHG2*	p.Arg1293Trp	Missense	19	0	Likely Deleterious	0.99	Likely Damaging
*RNF216*	p.Lys470Thr	Missense	7	0	Deleterious	0.992	Likely Damaging
*CNTN4*	p.Pro504Thr	Missense	3	0	Deleterious	0.994	Likely Damaging
*SFRP5*	p.Cys198Arg	Missense	10	0	Deleterious	0.997	Likely Damaging
*SKOR2*	p.Arg83Gly	Missense	18	0	Deleterious	0.997	Likely Damaging
*KIFAP3*	p.Glu90Lys	Missense	1	0	Deleterious	0.998	Likely Damaging
*SLIT1*	p.Arg471Cys	Missense	10	0	Deleterious	0.999	Likely Damaging
*PPFIBP1*	p.Arg105Met	Missense	12	0	Deleterious	0.999	Likely Damaging
*EFCAB2*	p.Arg87Ser	Missense and Splice Region	1	0.01	Deleterious	0.167	Benign
*NOD1*	p.Glu883Lys	Missense	7	0.01	Deleterious	0.205	Benign
*TFR2*	p.Ser756Phe	Missense	7	0.01	Deleterious	0.483	Likely Damaging
*SLC39A8*	p.Ser87Leu	Missense	4	0.01	Deleterious	0.71	Likely Damaging
*AFP*	p.Ala366Val	Missense	4	0.01	Deleterious	0.795	Likely Damaging
*ANK2*	p.Val2139Ala	Missense	4	0.01	Likely Deleterious	0.99	Likely Damaging
*MAST4*	p.His1466Asp	Missense	5	0.01	Deleterious	0.995	Likely Damaging
*CENPH*	p.Glu83Lys	Missense	5	0.01	Deleterious	0.996	Likely Damaging
*CKAP4*	p.Glu571Lys	Missense	12	0.02	Deleterious	0.082	Benign
*PAPOLG*	p.Asn551His	Missense	2	0.02	Deleterious	0.396	Benign
*ZNF141*	p.Ala72Pro	Missense	4	0.02	Deleterious	0.871	Likely Damaging
*CACNB1*	p.Ala60Val	Missense	17	0.02	Deleterious	0.996	Likely Damaging
*PRAMEF2*	p.Ser314Tyr	Missense	1	0.03	Deleterious	0.021	Benign
*AFP*	p.Ala225Thr	Missense	4	0.03	Deleterious	0.025	Benign
*TFAP2B*	p.His99Tyr	Missense	6	0.03	Deleterious	0.069	Benign
*PCLO*	p.Ala4060Val	Missense	7	0.03	Deleterious	0.254	Benign
*PALM*	p.Ala19Thr	Missense and Splice Region	19	0.03	Deleterious	0.64	Likely Damaging
*FCRL1*	p.Ala11Val	Missense and Splice Region	1	0.03	Deleterious	0.992	Likely Damaging

## Discussion

Most patients with intraepithelial EMPD achieve remission with an anticipated 5-year survival of over 60% [2]. However, when the disease invades the dermis, this percentage drops to 10%, with about one-third of patients developing lymph node involvement and distant metastases [2]. Spinal metastases in EMPD are rare but have been reported in a few cases. In two of such described cases, compression fractures in the vertebral bodies were described, and both cases resulted in patient demise within 5 to 6.2 months of spinal metastasis detection [36, 37]. Several studies have investigated the potential correlation between EMPD invasiveness and HER2 protein overexpression, although conclusive findings are hindered by small sample sizes. Invasive EMPD typically have a larger percentage of 2 + or 3 + HER2 IHC scores compared to in situ EMPD. However, IHC HER2 positivity ranges widely across reports between 18 − 71% and between 10 − 62% in invasive EMPD and in situ EMPD, respectively [7, 10, 12, 38, 39]. Therefore, despite serving as a useful biomarker, it cannot be assumed that HER2 positivity signifies EMPD invasiveness. In our case, the patient’s tumor was metastatic and exhibited strong IHC HER2 positivity. However, *ERBB2* presented minimal CNV gain from our CNV analysis. Previous studies have also reported IHC HER2 positive invasive EMPD cases with an absence of *ERBB2* gene amplification via FISH [8] and CISH [10]. While gene amplification detected by CNV gains using WGS cannot be directly compared against ISH methods, there seems to be a weak correlation between *ERBB2* gene amplification and HER2 protein overexpression. Exploring other signaling pathways associated with HER2 protein overexpression instead could be worthwhile.

The patient was administered with a unique EMPD treatment regimen combining HER2-targeting trastuzumab with paclitaxel together with denosumab, followed by the subsequent replacement of paclitaxel with anti-androgen bicalutamide. The patient exhibited a PR to this treatment for 7 cycles. A study by Sekiguchi et al. administered trastuzumab and paclitaxel combination chemotherapy as well. Of the four cases reported, one case exhibited PR with progression free survival (PFS) of 12 months, while the other 3 cases met with early disease progression [16]. Contrastingly, a study by Takahagi et al. administering the same combination chemotherapy eradicated HER2-expressing tumor cells in the patient’s dermal nests and lymph vessels 6 months into treatment. However, metastases in the CNS could not be reduced and eventually led to the demise of the patient [15]. In these studies, because the patients were previously treated with docetaxel, cross-resistance between docetaxel and paclitaxel was a concern [16]. The response of our patient was not influenced by any prior use of docetaxel.

Estrogen receptor, androgen receptor and progesterone receptor expression has been reported to be positive in 13%, 40% and 8% of patients with EMPD, respectively [40]. Previous EMPD treatments combining bicalutamide and leuprorelin with trastuzumab [2] and other anti-androgen drugs [41] have failed to prevent rapid disease progression. Only one case presented reduced bone and lymph node metastases upon bicalutamide and leuprorelin acetate treatment [42]. It has also been hypothesized that the androgen receptor overexpression might just be an inherent characteristic of glandular cells with apocrine differentiation [2]. With the limited anti-androgen treatments administered to EMPD, it is difficult to ascertain whether EMPD tumorigenesis depends on the androgen receptor signaling pathway. Finding an effective personalized treatment approach for each EMPD case remains a significant challenge even with the presence of certain biomarkers, and this is further complicated by the limited number of previous studies available as references.

An enrichment of genes on chromosome 8 associated with *TGFβ* was also reported. Although the role of TGFβ is not well studied in EMPD, a study by Hirakawa et al. [43] has investigated the onset of epithelial-mesenchymal transition (EMT) in EMPD, possibly enhanced by TGFβ. EMT is a process whereby polarized epithelial cells undergo biochemical remodelling to develop into mesenchymal cells, thus contributing to metastasis [44]. Our patient experienced metastases in the right iliac and inguinal lymph nodes, spine, and ribs, suggesting that lymphoangiogenesis and subsequent lymphatic invasions contributed to the metastatic spread. Histological examination of EMPD lesions have also revealed notable difference in the development and enlargement of lymphatic and blood vessels in the dermis compared to healthy skin or even other skin cancers including melanoma [43]. Previous studies have observed that invasive Paget cells demonstrated lower E-cadherin expression and higher expression of mesenchymal markers including N-cadherin and/or vimentin than normal epidermal keratinocytes and in situ carcinoma Paget cells, and the expression of EMT-related markers correlated with the incidence of lymphatic invasion in primary skin tumor [43, 45]. These findings substantiate the involvement of EMT-like mechanisms in the invasion of tumor cells in EMPD, particularly lymphatic invasion, and this is further enhanced by TGFβ. Additionally, some cell-based models have investigated the interaction between TGFβ and HER2 signalling pathways as well. For instance, TGFβ modulates the actin cytoskeleton, inducing cell migration and EMT in HER2-overexpressing breast cancer cells [44]. Exogenous TGFβ could also induces trastuzumab resistance in HER2-positive breast cancer cell lines [5]. Taken together, these findings highlight the crucial roles of HER2 and TGFβ in breast cancer metastasis. The functional crosstalk between TGFβ and HER2 could be of potential relevance to EMPD.

There was also an enrichment of the pathways associated with FGFR1 signalling. Fibroblast growth factor receptor (FGFR) is a target that has been explored as part of metastatic breast cancer therapies [46]. Various studies have shown that gene-translocations can lead to the expression of fusion proteins with FGFR, resulting to aberrant FGFR signalling which can act as oncogenic drivers of cancer [46, 47]. Despite limited research on the role of FGFR in EMPD, one study by Ishida et al. described that out of 87 cases, only 3 cases harbored *FGFR1* amplification [48]. Interestingly, the study postulated that amplifications or mutations in *ERBB2*, mutations in *ERBB3* and *FGFR1* amplification were mutually exclusive, suggesting that alterations in these 3 genes represents distinct disease subsets [48]. In our case, we observed HER2 overexpression via IHC and *FGFR1* amplification via notable CNV gains. Further research is necessary to establish the significance of our preliminary WGS data.

Among the genes that were showing notable CNV gains, *IDO1* and *IDO2* (log_2_ FC = 1.22256) were particularly interesting. IDO is an immunoregulatory enzyme; upon inflammatory stimulus, IDO converts tryptophan to kynurenine, inducing immunosuppression and inhibiting immune activation [49]. Similar to checkpoint molecules such as PDL1, cancer cells exploit these pathways to evade the immune system and suppress T-cell-mediated antitumor responses [49]. Promising IDO-inhibitors including indoximod (1-methyl-D-tryptophan) and epacadostat have shown promising results in clinical trials, although often in combination with chemotherapy and checkpoint inhibitors. Notably, there is a metastatic EMPD case reported with durable response to checkpoint inhibitor immunotherapy; 4 cycles of ipilimumab plus nivolumab treatment resulted in 7 months PR [50]. Collectively, these findings highlight the potential of future EMPD treatment regimens involving IDO inhibitors.

Among the genes with notable CNV loss, 5 tumor suppressors (*CDKN2A*, *CDKN2B*, *CUX1*, *LRP1B* and *PTPRD*) were identified. A prior EMPD study also revealed deletions of areas harbouring *CDKN2A* via CNV analysis [48]. *CUX1* was identified as a potential EMPD driver mutation, exhibiting deletions and truncating mutations in 19.5% of the EMPD cases studied, with 9.2% showing nonsense or splice-site mutations as well [48].. The paralleled findings suggest that the CNV loss of these tumor suppressor genes may have contributing roles to EMPD development.

## Conclusion

In conclusion, the presented case exemplifies the extrapolation of information from treating common cancers– in this case, breast cancer– to rare cancers by identifying their similar histological and molecular profile, thereby improving the treatment approaches. Despite being confronted with poor prognostic factors including spinal metastases, associated MAHA and HER2 positivity, the patient responded remarkably and survived for another 22 months post diagnosis under the administration of HER2-based treatment in combination with other therapeutic agents that were introduced sequentially over multiple treatment cycles. Through this case, both clinical information and genomic analysis were integrated to attain a more comprehensive understanding of the disease landscape.

### Electronic supplementary material

Below is the link to the electronic supplementary material.

Supplementary Material 1

## Data Availability

The data that support the findings of this study are available from the corresponding author upon reasonable request. The datasets supporting the conclusions of this article are available in the European Genome-Phenome Archive repository (accession number EGAS50000000243).
