# Supplementary material for: Whole genome sequencing of HER2-positive metastatic extramammary Paget’s disease: a case report
Source: Orphanet J Rare Dis. 2024 Jun 3;19:223. doi: 10.1186/s13023-024-03169-y (PMC11149212; doi:10.1186/s13023-024-03169-y)
Supplement: Supplementary file 1 — Supplementary Material 1 [file 13023_2024_3169_MOESM1_ESM.docx]

**Appendix I**

**OJRD-D-23-00911**

Associate Editor: The manuscript by Lim and coworkers reports the clinical, pathological and molecular findings in a single case of HER2-positive metastatic extramammary Paget’s disease. Both Reviewers considered the manuscript interesting and the study, overall, correctly performed. However, they also raised several comments that should all be addressed or at least adequately answered. In particular, the Authors should make clear in the manuscript title that the study refers to a single case and specify in the abstract background that the patient had a partial response to anti-HER2 treatment combined with other agents.

**AUTHOR REPLY:** We thank the Editor for the positive comments. We have addressed the issues in the point-by-point response below. In particular, we have made clear in the title that this is a case report, and that the patient had responded to HER2-directed treatment in combination with other agents.

Additional minor comments:

•	The “Introduction” section should be renamed “Background” to fulfil Orphanet J Rare Dis rules.

**AUTHOR REPLY:** In the revised manuscript, we have now renamed “Introduction” to “Background”.

•	Background section, line 63, sentence “… with an estimated prevalence ranging from 0.1 to 2.4 per million individuals.” The reference cited reports an estimated incidence (not prevalence) of 0.1 to 2.4 patients per 1,000,000 person-years. Can you please check?

**AUTHOR REPLY:** In the revised manuscript, we have amended the sentence in line 71 to “…with an annual incidence ranging from 0.1 to 2.4 new cases per million people.”

•	Background section, lines 63-64. “usually occurring in older persons aged 60 to 80 years old”. This part of the sentence would benefit from minor editing. The Authors might consider “usually occurring in elderly individuals aged from 60 to 80 years.”

**AUTHOR REPLY:** In the revised manuscript, we have now edited the sentence in line 70 to “EMPD usually occurs in elderly individuals aged from 60 to 80 years.”

•	Background section, lines 70-71. “… by eczematous lesions frequently presented on the nipple or areolar regions and is frequently linked to underlying in situ or invasive breast cancer…”. It might be preferable to write “… by eczematous lesions usually presenting on the nipple or areolar regions …”.

**AUTHOR REPLY:** In the revised manuscript, we have now amended “eczematous lesions frequently presented on the nipple or areolar regions…” to line 84: “eczematous lesions usually presenting on the nipple or areolar regions…”.

•	Results section, line 170. The expression “… which returned negative for malignancy.” is not frequently used. The Authors might consider: “… which were negative for malignancy.” or “… which gave negative results for malignancy.”

**AUTHOR REPLY:** In the revised manuscript, we have now amended in line 187 from “which returned negative for malignancy” to “all of which gave negative results for malignancy.”

•	Results section, Line 188. “Thereafter, he was then treated with capecitabine ...”. Using both “thereafter” and “then” seems redundant, the Authors might consider to simply write “Thereafter, he was treated with capecitabine …”.

**AUTHOR REPLY:** In the revised manuscript, we have now edited the sentence in line 204 to “Thereafter, he was treated with capecitabine…”.

•	Results section Lines 190-192. The sentence “At this juncture, the patient’s condition worsened through treatment about 22 months from diagnosis with rapid elevation of ferritin and LDH levels, leading to his eventual demise” is difficult to understand and would need improvement. The Authors might consider to simply state “Then, the patient’s condition worsened with rapid elevation of ferritin and LDH levels, leading to his eventual demise about 22 months after diagnosis.”, leaving considerations on the prolonged patient survival under treatment to the Discussion section.

**AUTHOR REPLY:** In the revised manuscript, we have now edited the sentence in line 206 to “Then, the patient’s condition worsened with rapid elevation of ferritin and LDH levels, leading to his eventual demise about 22 months after diagnosis.”

•	Discussion section, line 234. “… invades the derma …” should read “… invades the dermis …”.

**AUTHOR REPLY:** In the revised manuscript, we have now amended “derma” to “dermis” (line 249).

•	Discussion section, lines 236-237. “… compression fractures in the vertebral bodies was reported …”. In this sentence, “was” should read “were”, and to avoid repetitions with previous sentence (reported), it might be preferable to write “… compression fractures in the vertebral bodies were described …”.

**AUTHOR REPLY:** In the revised manuscript, we have now edited the phrase in line 252 from “was reported” to “were described”.

•	Fig. 1 legend, the sentence “Red arrows indicate as right iliac and inguinal lymph nodes indicated” is unclear. Did the Authors mean “Red arrows indicate right iliac and inguinal lymph nodes involvement” or something else?

**AUTHOR REPLY:** In the revised manuscript, we have now edited the phrase to “Red arrows indicate right iliac and inguinal lymph nodes involvement”.

•	References. According to Orphanet J Rare Dis rules for reference citation, a journal article can be cited either by reporting the publication year, volume and page numbers or by DOI. As your references report the publication year, volume and page numbers please omit the DOI.

**AUTHOR REPLY:** In the revised manuscript, we have omitted the DOI.

Reviewer #1: The manuscript "Whole Genome Sequencing of HER2-Positive Metastatic Extramammary Paget's Disease" is an interesting case report focused on the landscape of copy number variations revealed through whole genomic sequencing and has also potential therapeutic implications.

The work is complete and well-structured.

The design of the study is appropriate, and the images are satisfactory.

The language is acceptable.

It represents a good work, with the only limitation related to being a case report and should be expanded in a systematic series.

**AUTHOR REPLY:** We thank the reviewer for the thorough review of our manuscript and for the favourable comments. While our current work focuses on a single case report, we agree that a systematic series could provide additional insights and contribute to a deeper understanding of the topic. We will consider this suggestion for future research endeavours.

Reviewer #2: GENERAL COMMENTS

The title clearly describes the topic covered in the paper but I would include the reference of 'a case report'. This request is made in order to avoid the reader thinking that this is a prospective/retrospective study or a review of the literature.

**AUTHOR REPLY:** We thank the Reviewer for the comments. In the revised manuscript, we have added “a case report” to the title.

Regarding the case described, it might have been useful to repeat immunohistochemistry and genomics tests on metastases as well, to highlight any differences in expression between the primary tumor and the distant metastasis on a clinical point of view.

**AUTHOR REPLY:** We agree that it would be a valuable consideration to perform immunohistochemistry and genomics tests on metastases to compare expression differences between the primary tumor and distant metastases. We have performed immunohistochemistry for both the primary scrotal tumor and metastatic bone marrow sample – these results have been updated in lines 181 to 184: “Primary tumor cells were diffusely positive for CK7, CK20, Cam5.2 and negative for SOX10, TTF1, PSA, and CDX2. Metastatic tumor cells exhibited a similar staining pattern. Additional immunostaining on the metastatic tumor was positive for GATA3, androgen receptor and estrogen receptor, but negative for progesterone receptor.”. Genomic sequencing was not performed on the bone marrow sample, but we will certainly consider it for future studies.

Furthermore, the title focuses on the effectiveness of anti-Her 2 therapy but Trastuzumab is not the only therapy applied rather it was combined firstly with platinum and then with anti-androgen. I suggest to specify the interest in a possible therapeutic role of Trastuzumab in combination with other agents.

**AUTHOR REPLY:** We thank the reviewer for highlighting this point. While we understand that the title might give off the misleading impression that there is an exclusive focus on Trastuzumab, our primary interest lies in HER2 due to the strong HER2 protein overexpression in our case, as well as the significant expression of HER2 in EMPD. In the *Discussion* section, we have included discussion on the use of anti-androgen treatments in previous EMPD studies, demonstrating our inclusion and consideration of the other therapeutic options administered. In response to the reviewer's suggestion, we have revised the manuscript to highlight the combination of other agents with HER2-directed treatment, specifically in sections that indicated “HER2-directed therapy” only. This includes the *Background* and *Conclusion* section of the Abstract, the *Background* section, and the *Conclusion* section, where we have clarified the administration of other therapeutic agents as well.

Finally, a comprehensive linguistic review would be useful.

**AUTHOR REPLY:** We thank the Reviewer for bringing this to our attention. We have carefully reviewed the text and corrected any linguistic mistakes to ensure clarity and precision.

SPECIFIC COMMENTS

Introduction

Please specify that the EMPD is non only an intraepithelial carcinoma but that there are also micro- invasive and invasive disease, responsible of nodal and distant metastasis.

**AUTHOR REPLY:** We thank the Reviewer for the comments. In the *Background* section, we have included information on the invasive stage of the disease by adding “Although there is favourable prognosis for the early disease stage, in certain cases, it can advance to invasive EMPD. Invasive EMPD is characterized by infiltration into deeper tissues and is associated to nodal and distant metastasis.”

Please add literature references to better define the context and the actual knowledge on diagnosis, genomic and treatment options; in fact is not a new achievement to use anti-HER 2 molecules in specific selected cases (some examples - PMID: 36980691, PMID: 36731045, PMID: 31297606, PMID: 35084535, PMID: 37634315, PMID: 36980691)

**AUTHOR REPLY:** We acknowledge that the utilization of anti-HER2 therapy is not novelty; in the *Background* section, we have stated that “Such HER2-directed approaches have also been described in a few EMPD cases”. The challenge lies in the assessing the treatment efficacy of such HER2-directed approach in these select EMPD cases, which we mentioned in the *Background* section, and we highlighted the differing patient responses of previous antiHER2-treated EMPD studies in the *Discussion* section. Our study contributes whole-genome sequencing insights to the EMPD genomic landscape, expanding upon findings that have been described in current antiHER2-treated EMPD cases. In our revised manuscript, we have included the challenges and treatment options for managing EMPD under the *Background* section to provide more context on other available treatment options of EMPD.

Results

Please better define the histological examination. I think it is necessary to describe the disease as an invasive disease and not only intraepithelial.

**AUTHOR REPLY:** We thank the Reviewer for highlighting this point. We have updated the histology description to include the areas of invasive carcinoma seen in the specimen. Line 180 now include: “…, as well as regions of invasive carcinoma.”

Please indicate why did you completed the staging with the study of gastrointestinal tract and urinary cytology. It is known the possible association with other tumours. Please add literature references (some examples - PMID: 37793235, PMID: 38162507)

**AUTHOR REPLY:** In our revised manuscript, we have cited a study by Jones et al. (PMID: 232972) providing useful insights into the histological divisions of extramammary Paget's disease, including its involvement with contiguous epithelia of organs such as the genitourinary and gastrointestinal tracts. Our decision to conduct oesophagogastroduodenoscopy, colonoscopy, and urine cytology was guided by this understanding to comprehensively evaluate the patient's condition.

Line 192 Please substitute trajectory with strategy

**AUTHOR REPLY:** In the revised manuscript, we have substituted “trajectory” with “strategy”.

Discussion

Line 233 please specify intraepithelial/in situ EMPD.

**AUTHOR REPLY:** In the revised manuscript, line 248, we have specified “intraepithelial EMPD” as suggested.

Conclusion

The offered conclusions are adherent with the case described

**AUTHOR REPLY:** We thank you the reviewer for the comments regarding our conclusions. We appreciate the acknowledgment that the conclusions align with the details presented in the case.
